# The value of adding ‘grey literature’ in evidence synthesis for equity-driven global health: a case study from Small Island Developing States

**DOI:** 10.1136/bmjgh-2025-020906

**Published:** 2026-06-24

**Authors:** Anna Brugulat-Panés, Clara Martín-Pintado, Cornelia Guell, Nigel Unwin, Viliamu Iese, Eden Augustus, Louise Foley

**Affiliations:** 1 IMS Epidemiology Unit, University of Cambridge, Cambridge, UK; 2 European Centre for Environment and Human Health, University of Exeter Medical School, Exeter, UK; 3 School of Agriculture Food and Ecosystem Sciences, The University of Melbourne, Melbourne, Victoria, Australia; 4 Pacific Centre for Environment and Sustainable Development, The University of the South Pacific, Suva, Fiji; 5 Faculty of Medical Sciences, The University of the West Indies, Bridgetown, Barbados

**Keywords:** Global Health, Nutritional and metabolic disorders, Systematic review, Equity

## Abstract

Grey literature, defined as evidence ‘not controlled by commercial publishers’, can enhance the comprehensiveness, timeliness and balance of evidence reviews. It may be particularly important in global health research, where funding inequities and under-representation of local expertise in peer-reviewed research articles—often published by commercial publishers for substantial fees—can lead to evidence gaps in systematic reviews. However, searching, reporting and analysing grey literature poses conceptual and methodological challenges. We conducted a systematic scoping review of commercially published and grey literature examining food sources in Small Island Developing States (SIDS). The grey literature component aimed to expand understanding beyond built food environments and food retail and to promote epistemic justice by including traditionally excluded voices, knowledge and experiences. This might be especially important in SIDS, which, due to their small size, remote locations and limited global visibility, might not be well represented in commercially published literature. In this paper, we discuss how grey literature enriched our findings, offering detailed descriptions of diverse food sources, regional data and country comparisons less present in commercially published sources. These documents frequently reflected multisectoral initiatives and were developed through partnerships involving local stakeholders. Beyond highlighting the value of grey literature, we also provide practical guidance for its use in evidence synthesis. This includes key principles for effective searching and screening to support researchers aiming to incorporate grey literature in a systematic and transparent way. Our findings underline the importance of including grey literature to avoid excluding valuable perspectives and perpetuating structural imbalances in global health knowledge.

**Summary box**

- Evidence syntheses that rely primarily on commercially published literature may under-represent knowledge, experiences and research from less well-resourced settings.
- Using a systematic scoping review of food sources in Small Island Developing States as a case study, we show that grey literature substantially enhanced the comprehensiveness, inclusivity, diversity and timeliness of our research findings.
- Grey literature captured local perspectives, multisectoral initiatives and contextual information that were less visible in commercially published sources.
- Including grey literature should be considered not only a methodological choice but also an important component of equity-oriented global health research.

## Introduction

 There is an unprecedented growing momentum in global health to support more equitable systems free from residual colonial practices and thinking.^1^ Academics worldwide, particularly in Africa, are leading these discussions which aim to reshape current academic power structures.^2^,^8^ While this is relevant for many academic fields and disciplines, these power dynamics hold particular significance in global health research where imbalances persist between institutions from high-income countries and low and middle-income countries (LMICs).^5^

Inequities persist in the flow of research funding affecting access to resources, capacity building, research performance and subsequent publication.^2 9^ Publishing in peer-reviewed journals, controlled by commercial publishers, is a visible form of research productivity, central to a researcher’s career progress, affecting promotions and recognition. Publishing in international high-impact journals remains particularly challenging for researchers from less well-resourced institutions. This challenge stems from stringent gold standard criteria (based on dominant understandings of scientific rigour), unconscious biases from editorial boards and peer-reviewers in decision-making, and exorbitant open access article processing fees.^10 11^ These barriers exacerbate existing inequalities within the academic landscape. Similarly, mainstream literature often reflects paradigms, frameworks and methodologies shaped by privileged groups from rich, former colonising countries in Western Europe and North America.^12^ This prevailing narrative tends to overlook and undervalue other perspectives and scientific knowledge. This knowledge is frequently dismissed as lacking rigour and credibility, resulting in exclusion or underrepresentation in collective processes of knowledge production, interpretation and practice.^13 14^ Such instances constitute a form of epistemic injustice, referred to as the unfair discrimination of people against their capacity as knowers.^15^

Evidence syntheses are a key tool in public health to inform decision-making, and are increasingly common in other disciplines such as the environmental sciences.^16^ Acknowledging its importance, researchers have developed explicit methods for this form of research.^17^ These methods, rooted in principles of transparency, accountability and reproducibility, follow a comprehensive and systematic approach to evidence review, meticulously documented according to standardised reporting guidelines.^18^ However, previous studies have revealed that these methods do not fill the needs and capacities of researchers from LMIC institutions, leading to an underrepresentation of research conducted by these groups in bibliographic databases of evidence reviews.^19^

Various definitions exist for grey literature due to its diversity. However, certain characteristics are shared among all forms. For example, it includes information produced on all levels of government, academia, business and industry in electronic and print formats not controlled by commercial publishing^20^ (ie, publishing is not the primary activity of the producing body), it is difficult to find, its accessibility might be temporary, and it is not typically available from standard bibliographic databases.^21^ Grey literature includes both academic materials like theses and dissertations, as well as non-academic materials such as organisational reports and policy papers. In this study, we refer to grey literature as evidence ‘not controlled by commercial publishers’.^22^ While previous authors^23^,^27^ have emphasised the advantages of incorporating grey literature into evidence reviews, they have also highlighted the conceptual and methodological challenges associated with searching, reporting and synthesising such literature. Attempting to adhere to a systematic approach in reviewing grey literature can be laborious, time-consuming and challenging to replicate, due to the nature of this type of literature and the absence of an established method and comprehensive guidance. In addition, relevant policy documents, local reports and donor-funded evidence may remain offline or unpublished, further complicating efforts to comprehensively identify grey literature through online searches alone.

Evidence reviews serve as critical tools for identifying research gaps and effective interventions to inform policymaking.^17^ However, given the existing inequities, this paper contributes to the debate that solely relying on evidence from commercial publishers in evidence synthesis may present a limited perspective of existing research and, consequently, of what is most urgently needed. Implicit within this argument is the proposition that evidence reviews that exclude grey literature run the risk of overlooking local successful interventions and effective developments, resulting in a partial view for decision-making. In contrast, adding grey literature has the potential to enrich evidence synthesis by ensuring a greater representation of research conducted and led by local academics from less well-resourced settings. To address these challenges and underscore the importance of incorporating grey literature, we present a case study of a systematic scoping review examining food sources in Small Island Developing States (SIDS). Through this case study, we propose solutions and highlight the unique value of including grey literature in our research topic. In doing so, we aim both to demonstrate its value and to provide practical guidance on how grey literature can be incorporated into evidence reviews, ultimately translating our findings into a broader context and reaching a more diverse audience.

### The case study

SIDS are a heterogeneous group that share many commonalities.^28^ Most SIDS have become highly dependent on food imports mainly constituted by costly and unhealthy processed foods.^29^ This dietary shift, known as the nutrition transition, entails a departure from traditional diets rich in local produce to processed imported foods high in salt, sugar and fats.^30^ Consequently, SIDS have some of the highest rates of risk factors, morbidity and premature mortality attributed to chronic, non-communicable diseases (NCDs) globally.^31^ The food systems of SIDS face important challenges influenced by colonial legacies, trade liberalisation agreements and climate change.^32^ As a result, SIDS have implemented robust national policies and strategies focused on NCDs prevention and long-term resilience, also positioning themselves as leaders in global climate justice initiatives, particularly on issues of funding for loss and damage.^33^ Given SIDS complexities, understanding the various types of food sources driving and influenced by food production and consumption is important to advancing the UN Sustainable Development Goals.^28^ To address this need, we conducted a systematic scoping review including both commercially published and grey literature on food sources in SIDS.^34^

By adding grey literature in our evidence review, we aimed to capture food sources and practices beyond physical (built) food environments such as supermarkets, which are often the focus of commercially published literature on this topic. As noted by previous authors,^35^ this focus could be due to an imbalance of research on urbanised and market-driven settings where more heterogeneous food sources such as wild foods are less common. Similarly, we aimed to include food sources characterised by small-scale economies, driven by socio-cultural livelihoods and with a strong and well-developed habitus of informality that differ from Western-type food sources and therefore could be overlooked in mainstream literature. Finally, we also aimed to capture local strategies and experiences concerning food sources in SIDS which, given their generally small size, remote locations, developing status and a total population comprising less than 1% of the global population, might be underrepresented in commercially published literature and instead more prominently reflected in grey literature such as reports, working papers and newsletters.

### How-to: grey literature searching and screening

We included grey literature documents published between January 1992 and June 2021, without restrictions on language or study type. Searches were conducted between 11 June 2021 and 25 June 2021. Eligible documents were those reporting on the variety, extent or nature of one or more food sources within SIDS. We excluded whole books (but not single book chapters) and theses considered not relevant to the mapping purpose of the scoping review, as well as conference proceedings or abstracts, due to providing insufficient information for extraction. All other grey literature formats were eligible for inclusion. Full search strategies, detailed inclusion and exclusion criteria and citations of included documents are reported in our overarching publication^34^ and in the registered study protocol in Open Science Framework (https://doi.org/10.17605/OSF.IO/7ME4R). Searches were conducted in online repositories of UN agencies, websites of international and regional networks and bibliographic databases that index grey literature (see table 1 for specific platform details).

**Table 1:** Sources of grey literature included in the review

Source type	Description	
Online repositories of UN agencies	WHO IRIS	Documents and publications from HQ and Regional Offices, WHO periodicals, articles outside periodicals on WHO work, governing bodies documents, press and audiovisual materials, historical materials. Contains WHOLIS database.
	PAHO IRIS	Locally produced information. South–South information sharing.
	FAO Document Repository	Publications, reports and other papers and documents published by FAO, as well as some materials produced by other sources with FAO involvement.
	AGRIS Database	Supported by FAO. Food and agricultural scientific research database with special attention to scientific information produced in the global south.
	WFP Resources and Data	Repository of publications, annual reports and other papers and documents published by WFP.
	IFAD Knowledge Repository	UN agency focusing on poverty and hunger in rural areas of developing countries. Repository including publications, reports, other papers and documents.
	UNICEF Research and Reports Repository	Repository of publications using household surveys, annual reports and stories organised by topic including child health and child nutrition.
	UNOPS Resources Repository	UN agency dedicated to implementing infrastructure and procurement projects for the UN System, international financial institutions, governments and other partners around the world.
	UN OHRLLS Resources Repository	Publications, meetings and official documents related to Small Island Developing States.
Websites of international and regional networks	Hungry Cities Partnership	International network of cities and city-based partner organisations which focuses on the relationships between rapid urbanisation, informality, inclusive growth and urban food systems in the Global South. Includes reports, papers, book chapters, articles, presentations, briefs, theses.
	RUAF	Global partnership on sustainable urban agriculture and food systems.
	IIED	Publications including briefings, case studies, reports. Includes publications from the journal *Environment & Urbanization*.
	ShareCity Database	A collaborative and transdisciplinary approach to assess the practice and sustainability potential of city-based food sharing economies. Publications, presentations, blog articles.
	Caribbean Agribusiness	A website containing information on agri-food systems in the Caribbean region.
	The Pacific Community, formerly the SPC	A central repository, online cataloguing and dissemination system of Pacific Island statistical microdata, metadata, reports and documents.
	CARPHA	Publication catalogue including annual reports, manuals, guidelines, newsletters and NCDs and nutrition-related publications.
	The Conversation	A network of not-for-profit media outlets publishing news stories and research reports online, with accompanying expert opinion and analysis. Articles are written by academics and researchers under a Creative Commons licence, allowing reuse without modification.
Bibliographic databases inclusive of grey literature	Web of Science, SCOPUS, EconLit, AGRICOLA, PsycINFO and PubAg	Databases including conference proceedings, book series, book chapters, books, working papers, dissertations, monographs, serials, audiovisual materials and non-peer-reviewed articles.
In-country topic experts	Colleagues at the University of West Indies	
	Colleagues at the University of the South Pacific	

AGRIS, International System for Agricultural Science and Technology; CARPHA, The Caribbean Public Health Agency; FAO, Food and Agriculture Organization; IFAD, International Fund for Agricultural Development; IIED, International Institute for Environment and Development; IRIS, Institutional Repository for Information Sharing; NCDs, non-communicable diseases; PAHO, Pan American Health Organization; RUAF, Resource Centre on Urban Agriculture and Food Security; SPC, South Pacific Commission; UNICEF, The United Nations Children's Fund; UN OHRLLS, The UN Office of the High Representative for the Least Developed Countries, Landlocked Developing Countries and Small Island Developing States; UNOPS, The United Nations Office for Project Services; WFP, World Food Programme; WHO, World Health Organization; WHOLIS, World Health Organization Library Information System.

As recommended by others,^36 37^ we used a variety of different methods to conduct our evidence search, as outlined in table 1. In order to identify relevant sources of evidence, we consulted two experienced medical librarians and local research partners, building on our approach from a previous study.^38^ Guided by the focus of our review and the rationale behind including grey literature, we searched in nine publication repositories of UN agencies and eight website search engines of key international networks. Additionally, we searched in six bibliographic databases tailored for commercial publications but inclusive of grey literature. In-country based research partners also helped to identify relevant documents we could have missed from these searches. We did not include Google or other general search engines, as our targeted repositories, databases and in-country partner–identified sources provided comprehensive coverage within available time and resources. From consultation with an experienced librarian in a previous unrelated systematic review, we also understand that Google’s algorithmic ranking and users’ prior search activity can influence the results retrieved, making search standardisation and replicability even more challenging for this source than for other grey literature repositories.

We established a set of key terms covering the concept of food sources within the context of SIDS drawing on search terms used in our previous research study.^39^ As recommended by others,^23^ we kept broad and simple terms, agreed on with the research team, to ensure maximum sensitivity. We adopted different searching and browsing approaches depending on the source as they required different techniques. For example, while some sources provided a search function allowing the direct input of our key terms, others required manual navigation through headings or scanning lists of publications. Details on the key search terms can be found in online supplemental file 1 of a related publication.^34^

Following existing guidance,^40^ we documented the search process with enough detail throughout the process to ensure its transparency and facilitating reproducibility to a certain extent. We used an Excel database to record details on website name, URL, date of search, section where the search was conducted, search terms used, number of results retrieved, and number of results included. Accurate recordkeeping helped us to keep a consistent approach across similar resources, while it gave the flexibility to adapt our search strategies as needed. Details on the search process can be found in online supplemental file 1 of a related publication.^34^

**Figure 1:**
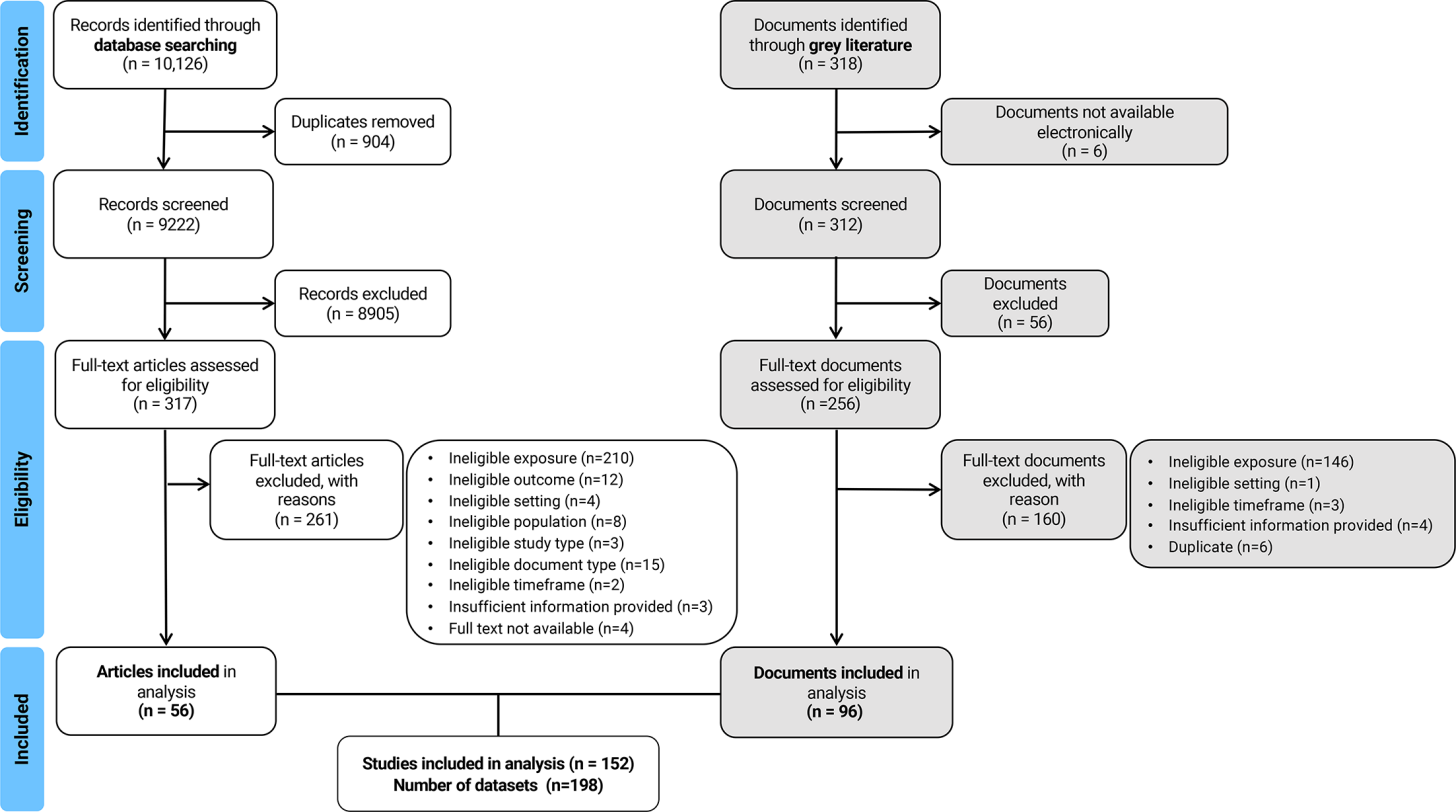
Preferred Reporting Items for Systematic Reviews and Meta-Analyses flow chart for grey literature included in the review.

We conducted document screening using an Excel database and Google Drive as a repository of selected documents. We adapted existing standards of rigour for document eligibility: we piloted a subset of retrieved documents at various screening stages. Two independent researchers undertook blind double screening of all documents, with conflicts resolved by a third team member. When downloading or export in bulk of documents was not available, we took a pragmatic approach of single screening ‘on screen’ for relevant documents. For large documents (more than 100 pages), full-text screening was conducted using the 'Table of Contents’ or the 'Search tool’ to identify and focus on relevant sections. A shared list of keywords facilitated consistency among reviewers in locating these sections. Only documents meeting eligibility criteria were compiled in an Excel workbook, where data charting took place. Reviewers kept reflexive notes during the data charting process, including document characteristics and author reflections. Ten percent of documents were double charted. This method enabled us to produce a flow diagram adhering to the Preferred Reporting Items for Systematic Reviews and Meta-Analyses, adding transparency and rigour in our reporting (see figure 1). We provide in this article an additional online supplemental file containing the data extraction templates and guiding notes used for both grey literature and peer-reviewed datasets.

Based on our experience with this case study, we present a set of core methodological principles for conducting grey literature searching in box 1.

Box 1 **Core principles of grey literature searching**

**Prioritise transparency over reproducibility**

- Emphasise transparency throughout the research process, particularly when documenting the search methodology. Acknowledge the inherent challenges in replicating grey literature findings and emphasise transparency as fundamental to valuable research.

**Thoughtfully develop and bound a search strategy**

- Develop a search strategy purposefully and thoughtfully, guided by your research questions and the rationale behind including grey literature. Carefully collate grey literature sources with input from method experts, topic specialists, and local stakeholders to ensure relevance and comprehensiveness.

**Rigour, applied flexibly**

- Apply existing conventions of rigour in systematic reviews thoughtfully and flexibly while navigating the complexities of grey literature and being mindful of potential implications for perpetuating epistemic injustices.

### Unique added value of grey literature

Our review included 152 studies and was constituted by 198 data units, as some studies were multicountry studies. Our analysis included 56 studies sourced from commercial publications and 96 studies from grey literature (figure 1). The substantial volume of grey literature documents meeting our inclusion criteria, in comparison to the commercially published dataset, underscored the importance of this body of literature as a source of information within our topic.

**Figure 2:**
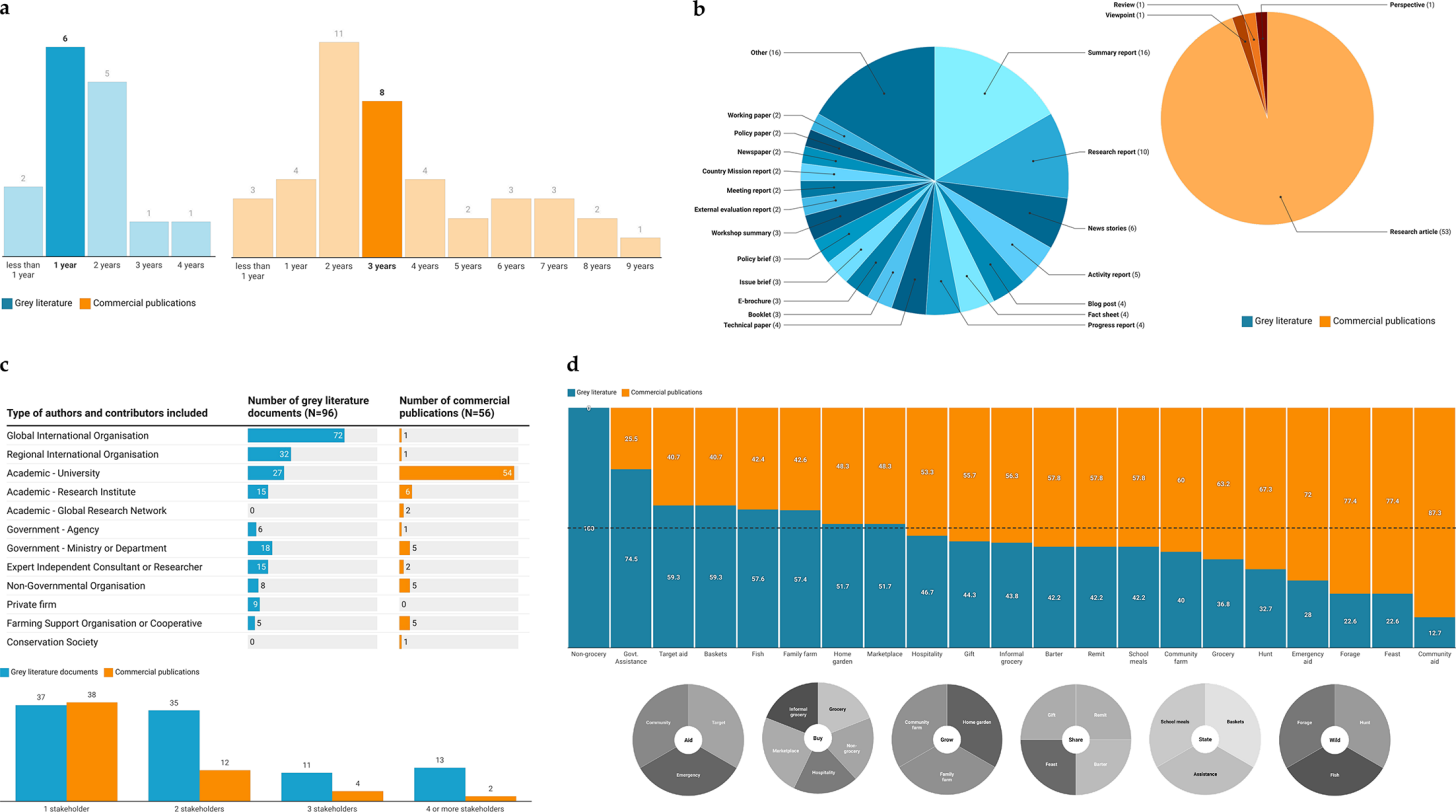
Comparison of characteristics between grey literature and commercial publications. (**a**) Histograms show the distribution of grey literature documents (blue) and commercial publications (orange) by the year gap between reported data collection and study publication dates. Grey literature (N=15): Mean=1.53 years, Median=1 year. Commercial publications (N=41): Mean=3.42 years, Median=3 years. In cases of multiple data collections (eg, several fieldwork visits), the latest data point was used for analysis to ensure consistency among all studies. (**b**) Pie charts depict material types found in grey literature (N=96, blue) and commercial publications (N=53, orange), with study numbers in parentheses. Material types were recorded as described by the study’s publisher; otherwise, they were categorised according to the Grey Literature Network Service live-list.^50^ The ‘Other’ category comprises materials such as country assessment reports (2), regional assessment reports (2), case study reports (2), synthesis reports (1), seminar proceedings (1), roundtable reports (1), reviews (1), regional summary reports (1), magazine articles (1), guidelines (1), formative research studies (1), city profiles (1) and book chapters (1). The ‘Research article’ category includes terms like original article, research article, article, original paper and research, as used by the publishers. (**c**) Table displays different types of authors and contributors and the number of studies that included them in each dataset. Authors and contributors were identified based on the affiliations reported to participate in the studies. ‘Government agency’ refers to specialised bodies with specific functions, narrower than those of the ‘Government Ministry or Department’. The column chart illustrates the number of grey literature documents (blue) and commercial publications (orange) that incorporated combinations of 1, 2, 3, 4 or more stakeholders in their studies. (**d**) Stacked bar chart depicts the percentage of studies within each dataset reporting various food sources, with a dotted line indicating the 50% mark. Food sources were identified based on our proposed classification specific to the SIDS context, as shown in the accompanying multiple pie charts. The classification includes Aid, Buy, Grow, Share, State and Wild, where Aid (foreign funded), Buy (purchased), Grown (locally produced), Share (exchanges), State (government funded), Wild (natural). ‘Non-grocery’ refers to pharmacy, bookstore, gas station, internet retail and door-to-door retail.^34^ NGO, non-governmental organisation; SIDS, Small Island Developing States.

Full extraction of research details such as study design, data collection methods or tools was not feasible for all grey literature documents as these aspects were either not reported or considered not relevant based on the type of evidence presented. This suggests that grey literature could serve as a platform for disseminating valuable research that may not meet the reporting standards required by commercial journals. In contrast to commercial publications, some grey literature documents provided insightful reflections on the barriers encountered during research and presented null findings, indicating a potential reduction in publication biases within this platform. Furthermore, we observed a variety of timeframes between data collection and publication years in grey literature documents, with a mean year gap of 1.5 and a median of 1 year gap. In comparison, commercially published studies presented a longer mean year gap of 3.4 years and a median of 3 years. It is worth mentioning that this analysis included a larger dataset of 41 commercial publications compared with 15 grey literature documents. This discrepancy arose because not all grey literature documents had data collection timelines that aligned for direct comparison with the research articles from the commercially published dataset. Nonetheless, these findings highlight the potential for grey literature to offer a pathway for timely dissemination of relevant research that could be delayed by commercial publishing or at least by certain high-impact journals (figure 2a).

Among the 96 included grey literature documents, we identified 32 distinct types of materials, each with diverse characteristics in terms of format and content. This stands in contrast to the standard format for research articles mostly found in commercial publications (figure 2b). The range of grey literature documents illustrated the heterogeneity of existing evidence and provided a more inclusive platform for publishing various research types and findings than the commercial publishers.

Regarding the geographic distribution of the included grey literature, we found representation of countries from all three SIDS regions—the Atlantic, Indian Ocean and South China Sea, Caribbean and Pacific regions. These documents provided insights into food sources at regional (12.6% of total datasets), national (49.5%) and city levels (3.5%), and included country comparisons (13 documents), dimensions that were less or not covered in the commercially published studies (0.5%, 33.9%, 0% and 6 publications, respectively). These descriptions, more frequently found in grey literature, enriched our understanding of the topic within SIDS both as a group and as individual settings. On average, documents from grey literature involved a greater number of different disciplines in their studies (mean=2.7, median=3) compared with the commercially published dataset (mean=2.3, median=2). The grey literature documents also included a wider range of authors and contributors, collaborating to our review by providing rich contextual understanding (figure 2c).

Regarding the types of food sources reported, both commercial publications and grey literature covered a wide range. Proportionally, grey literature more frequently documented food aid, state food sources, own grown and wild food sources such as government assistance programmes, food baskets, family farming and subsistence fishing. Interestingly, informal food sources such as street vendors and corner shops, and food sharing practices such as feasting were proportionally reported more often in commercial publications, contrary to our initial expectations (figure 2d). However, the eligible dataset included nearly twice as many grey literature documents as commercial publications (N=96 vs N=56), suggesting that valuable information on these informal food sources and practices could have been overlooked if grey literature was not included.

## Conclusions

Our case study focuses on SIDS, and its findings should be interpreted within this context. While SIDS span low- to high-income classifications, results may differ in non-SIDS settings, with the contribution of grey literature being context-dependent. This study illustrates the value of including grey literature in SIDS; we suggest that added value will be found in other settings, with the nature of that added value being context-dependent.

Adding grey literature into our evidence review enhanced the comprehensiveness, inclusivity, diversity and timeliness of our research findings. Grey literature also broadened the scope of our understanding by capturing a breadth of populations, places and publishers. Specifically, grey literature documents offered insights from various geographic locations within SIDS at regional, national and city-level. They also encompassed research conducted by multidisciplinary teams and a wide range of institutions, including governmental and non-governmental organisations, academic institutions and community-based organisations, providing a comprehensive perspective on our topic. By involving local authors and contributors, grey literature provided a nuanced understanding of complex issues and ensured representation from less well-resourced settings. Without the inclusion of grey literature, our review would have presented an incomplete picture. For example, we would have missed locally authored reports documenting implementation barriers, small-scale programme evaluations and context-specific policy actions, all of which were essential to understanding food sources across SIDS.

Failing to incorporate grey literature in evidence synthesis risks omitting important research findings and undermines efforts to address pressing global challenges effectively. However, it needs to be noted that including grey literature can be labour and time-intensive. Its inclusion might require resources that not everybody has access to, thereby creating an equity issue itself. Moreover, some forms of policy-relevant evidence in SIDS remain offline or circulate only through local institutional networks, meaning that even extensive online searches may fail to capture them. Future studies may therefore benefit from complementing online approaches with consultation of local officials, sectoral experts and in-country stakeholders—an approach that often relies on sustained relationships and local research leadership, further highlighting the importance of equitable partnerships.

When searching grey literature, caution is required due to the lack of consensus on rigour and quality compared with peer-reviewed literature. Limitations include the difficulty of identifying all relevant items, bounding searches and synthesising information when items vary in purpose or quality. Several authors have highlighted the specific appraisal challenges posed by grey literature, and practical tools such as Tyndall’s quality appraisal checklist provide structured ways to assess credibility, transparency and relevance.^41^ We propose that equity is also a key consideration, particularly whether documents capture lesser-heard voices or under-represented local perspectives often absent from commercial publishing. Approaches that recognise diverse ways of knowing—such as storytelling, local knowledge and Indigenous perspectives—can further enhance the trustworthiness and contextual relevance of the evidence.^42^ As highlighted in summary box 1, searches should be guided by an overarching spirit of enquiry and specific research questions, with explicit consideration of sources most likely to provide relevant insights. Transparency is key for replicability; documenting search decisions, sources and engagement with method experts, topic specialists and in-country stakeholders helps ensure that grey literature searches are as systematic and reproducible as possible.

By highlighting the structural inequities inherent in global health research and advocating for the inclusion of grey literature in evidence reviews, this study contributes to ongoing discussions concerning research inequalities. Our findings offer valuable insights for researchers, policymakers and practitioners across various fields and disciplines, who aim to adopt more equitable approaches in their projects. We provide additional recommendations that could contribute to this aim including a consideration of open-access journals with no processing charges such as Diamond Open,^43^ Research Directions^44^ and WHO Bulletin.^45^ Embracing initiatives like The Declaration on Research Assessment^46^ could also contribute to more equitable practices, as well as initiatives on citation justice recognising BIPOC (Black, Indigenous and People of Colour) groups or leaders in relevant topic areas.^47^ The use of tools such as ‘Connectedpapers’^48^ can facilitate the identification of BIPOC leaders in academia. Finally, reflecting on one’s positionality as a researcher through tools like the 'Social Identity Map’^49^ can enhance awareness and promote inclusivity in academia.

Using grey literature meaningfully demands not only methodological transparency, enquiry-driven search strategies, careful appraisal and a reflexive approach to rigour, but also equitable collaboration to access locally held knowledge that may lie beyond formal or online systems.

## Supplementary material

10.1136/bmjgh-2025-020906 online supplemental file 1

## Data Availability

All data relevant to the study are included in the article, its supplementary materials, the registered study protocol (https://doi.org/10.17605/OSF.IO/7ME4R), or the overarching publication [34].
